# Training the trainers: a survey of simulation fellowship graduates

**Published:** 2017-06-30

**Authors:** Patrick G. Hughes, Jose Cepeda Brito, Rami A. Ahmed

**Affiliations:** 1 Charles E. Schmidt College of Medicine, Florida Atlantic University, Division of Emergency Medicine, Florida US; 2 Summa Akron City Hospital, Department of Emergency Medicine, Department of Medical Education, Ohio, US

## Abstract

**Background:**

Coupled with the expansion of simulation has been the development and growth of medical simulation fellowships. These non-accredited fellowships do not have a standardized curriculum and there are currently no studies investigating the simulation fellowship experience. The purpose of this study was to explore the simulation fellowship experience of graduates throughout North America and how it prepared them for their post-fellowship career.

**Methods:**

A web-based survey was developed by Emergency Medicine attending physicians both of whom completed one-year fellowships in medical simulation. Prior to distribution, the survey was reviewed and tested by three simulation fellowship graduates and a PhD researcher. Feedback was integrated into the survey prior to distribution. The survey consisted of a maximum of 29 multiple choice questions including two step-logic questions and two open response questions. The survey was distributed to simulation fellowship directors in multiple disciplines and the directors were asked to forward the survey to graduates. Additionally, the Society for Academic Emergency Medicine Simulation Academy list-serve was utilized for distribution of the survey.

**Results:**

The survey had 35 responses. The majority of respondents completed fellowship within the last two years (66%, 23/35). Fellowship graduates strongly agreed or agreed that their fellowship adequately prepared them for their post-fellowship simulation career (88%). Graduates report that research design/reporting (53%) and administration (18%) were areas of their fellowship curriculum that needed the most improvement.

**Conclusion:**

The majority of simulation fellowship graduates agreed that their fellowship experience adequately prepared them for their post-fellowship simulation career. Graduates also felt that training in research and administration are areas that could be improved.

## Introduction

Simulation is an expanding method of instruction in medical education.^1^ This growth is a result of multiple factors including the effectiveness of simulation teaching methodology,^2^–^6^ increasing popularity with learners, and the greater availability of simulation equipment and centers.^7^–^9^ Coupled with this expansion has been the development and growth of medical simulation fellowships.^7^,^10^ Simulation fellowships, in multiple healthcare disciplines, provide education and training so that graduates can effectively lead simulation exercises and provide administrative leadership. Instruction typically includes adult-learning theory, assessment methodology, principles of debriefing, simulation scenario design, and the utilization of high-fidelity simulators and equipment.^10^ Over the last 10 years, the number of fellowships offered has increased tenfold.^10^

Fellowship training offers numerous benefits. Fellowship trained physicians in other disciplines have shown improved career satisfaction, a greater number of publications, an improved likelihood of obtaining grant funding, and an easier path to academic promotion.^11^ Fellowship trained physicians report feeling more prepared for their career endeavors.^12^,^13^ Additionally, fellowship training provides the foundation for long term mentoring, essential to the success of young faculty as they establish their careers in academia.^11^ Medical simulation fellows, regardless of discipline, appreciate those same benefits and have a number of unique opportunities to improve patient safety,^14^,^15^ the quality of medical education,^16^ and related research at their respective institutions.

Medical simulation fellowships are currently not accredited by the Accreditation Council on Graduate Medical Education (ACGME) or the Royal College of Physicians and Surgeons of Canada (RCPSC). The majority of these non-accredited fellowships do not have a standardized curriculum, likely leading to varied experiences by fellows. Graduates of these programs hold important roles within academic institutions and the training of these individuals as educational leaders has a substantial impact within their communities. There are currently no studies investigating the simulation fellowship experience from the perspective of fellowship graduates. The purpose of this study was to explore the experience of simulation fellowship graduates throughout North America and how the fellowship prepared them for their post-fellowship career.

## Methods

### Study design and participants

A web-based survey (www.surveymonkey.com) for simulation fellowship graduates was constructed and distributed electronically in the spring of 2015 to 28 simulation fellowship directors in the United States and Canada with up to four reminders in a two-month period. The simulation fellowship program directors were asked to distribute the survey to graduates of their programs. In order to identify simulation fellowship programs, a web-based search was performed using internet based search terms including, “simulation fellowship,” “medical simulation fellowship,” “surgical simulation fellowship,” and “anesthesia simulation fellowship.” Additionally, the Society for Academic Emergency Medicine (SAEM) Simulation Academy list-serve was utilized for distribution of the survey because of the significant number of simulation fellowships based in emergency medicine.^10^ The exact number of simulation fellowship graduates is unknown. However, previous scholars have estimated that there are approximately 55–60 simulation fellowship graduates as of 2015. Additionally, there are now approximately 25 graduates annually in North America.^17^ At the time of this study no database of simulation fellowship graduates existed. The correspondence through the list-serve indicated that the survey was exclusively for simulation fellowship graduates. The Summa Institutional Review Board granted an exemption for this anonymous research study.

### Data-collection methods and analysis

The survey was developed by two Emergency Medicine attending physicians who both completed one-year fellowships in medical simulation. Kern’s curriculum development provided the conceptual framework for questions concerning the simulation fellowship curriculum including needs assessment for targeted learners, goals and objectives and evaluation and feedback.^18^ In addition, the survey questions that focused on feedback, curriculum integration, skill acquisition, transfer to practice and instructor training were based on McGaghie et al.’s 12 features and best practices of medical simulation.^19^ Prior to distribution, the survey was reviewed and tested by three simulation fellowship graduates and a PhD researcher. Feedback was integrated into the survey. The survey consisted of a maximum of 29 multiple choice questions including two skip-logic questions and two open response questions.

Survey results were analyzed using Microsoft Excel. Frequencies, means, and ranges were calculated as appropriate*.* All submitted survey responses were incorporated in the analysis, including responses from incomplete surveys.

## Results

The survey had 35 responses from simulation fellowship graduates; the total number of simulation fellowship graduates is unknown. Twenty-eight of the 35 responses were from simulation fellowship graduates responding to the forwarded email from their fellowship directors while the remaining seven responses were from the SAEM list-serve. Thirty-three of the 35 respondents that started the survey completed the entire survey. This resulted in 33–35 responses per question.

### Fellow characteristics

Eighty-nine percent (31/35) of respondents were 25–44 years old and 53% (18/34) were male. The majority of respondents completed fellowship within the last two years (66%, 23/35). Seventy-seven percent (27/35) completed a one-year fellowship. The most common specialty was emergency medicine (71%, 25/35) (Table 1).

### Simulation fellowship experience

When reporting work time distribution, most fellows spent 11–20 hours per week on simulation fellowship responsibilities (36%, 12/33). In comparison, 62% (21/34) of fellows spent 11–20 hours per week on clinical responsibilities. Most respondents felt they had the ability to debrief effectively with minimal guidance after 4–6 months (44%, 15/34) and to run a high-fidelity full body simulator within 3 months (59%, 20/34) (Table 2).

### Advanced degrees and scholarship

Seventy-six percent (26/34) completed at least one national presentation or abstract during fellowship (1 presentation - 32%, 11/34; 2 presentations – 29%, 10/34; 3 presentations – 15%, 5/34), while only 50% (17/34) submitted at least one full manuscript (1 manuscript – 11/34; 2 manuscripts –5/34; 3 manuscripts – 0/34; 4 manuscripts – 0/34; ≥5 manuscripts –1/34). Thirty-two percent (11/34) of fellows pursued a master’s degree with 91% of them (10/11) choosing education.

### Post-fellowship evaluation

The majority of graduates strongly agreed or agreed that they had adequate faculty mentorship (88%, 30/34). Fellowship graduates strongly agreed or agreed that their fellowship adequately prepared them for their post-fellowship simulation career (88%, 30/34). Fellowship graduates strongly agreed that debriefing training during fellowship prepared them to conduct debriefing independently (76%, 26/34). In comparison, only a minority strongly agreed that the research (24%, 8/34), administration (21%, 7/34) and simulation technology (26%, 9/34) training prepared them for their post-fellowship simulation career (Table 3). When evaluating their fellowship experience, fellows felt the most challenging part of their fellowship curriculum to master was research design/reporting (68%, 23/34), followed by teaching and debriefing learners (18%, 6/34). Graduates reported that research design/reporting (53%, 18/34) and administration (18%, 6/34) were areas of their fellowship curriculum that needed the most improvement.

### Post-fellowship employment

First appointment post-fellowship: 26% (9/34) of the graduates were simulation directors and 21% (7/34) were simulation assistant directors while 29% (10/34) had a non-leadership simulation faculty appointment. Of the graduates working in simulation, 46% (13/28) are simulation directors with 71% (20/28) of graduates working in university/academic simulation centers and 18% (5/28) working in community hospital affiliated simulation centers. A few of these graduates may either be working in their second appointment or did not originally secure a simulation position straight out of fellowship. While 85% (28/33) of graduates work in simulation, 29% (8/28) of these have no protected time for simulation education and therefore do not receive salary for these simulation activities.

## Discussion

This survey provides a glimpse into the experiences of simulation fellowship graduates and how their fellowships prepared them for their careers. Two-thirds of surveyed fellowship graduates completed fellowship within two years of the survey. This is consistent with the recent growing popularity and expansion of simulation fellowships.^10^ As simulation becomes more prevalent in education, the demand for faculty with simulation training will continue to increase.

More than two-thirds of fellows completed a one-year fellowship. However, other respondents completed a fellowship that was less than a year in length or even two years in length. The variability in the duration of simulation fellowships is secondary to the lack of a standardized curriculum for simulation fellowships. Without a consensus, curriculum simulation fellowship directors independently determine the goals, objectives and length of their respective fellowship programs leading to variations in fellowship duration and content. This variability highlights that fellowships, each with their own goals and objectives, provide a range of experiences for fellows. Fellows may choose a program that concentrates on virtual reality, educational research, or standardized patient simulation. The lack of standardization allows fellows the opportunity to focus on their own interests in this growing body of knowledge about this teaching methodology.

More than two-thirds of graduates were emergency medicine (EM) physicians. EM residency programs make heavy use of simulation education and this may lead more residents to pursue a fellowship in simulation. Additionally, emergency medicine residency programs graduate more residents than surgery or anesthesia programs.^20^

During fellowship, most graduates felt they had the ability to debrief effectively with minimal guidance after 4–6 months and the majority of fellowship graduates felt they had the ability to run a high-fidelity full body simulator within 3 months. This is consistent with the majority of simulation fellowship graduates who either strongly agreed or agreed that debriefing training and simulation technology training during fellowship adequately prepared them for their post-fellowship career. Debriefing, as well as simulation technology training, are vital to being an effective simulation educator post-fellowship. Without these skillsets, especially debriefing, fellowship graduates would have difficulty effectively educating learners using simulation-based medical education. These results are mostly consistent with the 2015 Ahmed survey of simulation fellowship directors, which reports that fellows typically require 4–6 months before they are able to effectively debrief or run a high-fidelity full body simulator with minimal guidance.^17^

The majority of graduates felt the two biggest areas in need of improvement were research design/reporting and administration. This finding is consistent with the fact that only half of fellowship graduates reported completing of a manuscript during their fellowship. Additionally, this may contribute to the reason nearly one-third of fellowship graduates take positions that do not have any salary support. Future simulation fellowship curriculua should incorporate research and administration training to provide graduates with the tools necessary to be successful in their simulation career.

This study has several limitations. The total number of simulation fellowship graduates is unknown and while the survey was distributed to simulation fellowship directors from multiple specialties in the United States and Canada, the survey was only distributed to one specialty’s list-serve for simulation with a known high number of fellowship graduates (emergency medicine).^10^ There is no one established list-serve for all simulation fellowship graduates. Therefore, the survey was distributed to fellowship directors to forward to their graduates. Directors who did not forward the survey limited our ability to receive data from fellowship graduates. Additionally, there is no comprehensive list of fellowship directors. Those programs that did not have an internet presence or up-to-date contact information were unlikely to forward the survey to their graduates. The generalizability of the study is limited because the survey received only 35 responses from 28 fellowship programs and one list-serve. The survey was anonymous and therefore impossible to compare the demographics of those who completed the survey to non-responders. Furthermore, the number of programs has grown since the survey, potentially decreasing the representativeness of these findings.

### Conclusion

Simulation fellowships are a rapidly expanding area of medical education. The majority of simulation fellowship graduates agreed that their fellowship experience adequately prepared them for their post-fellowship simulation career. However, graduates felt that training in research and administration are two areas that could be improved. This survey provides valuable information and insight into the simulation fellowship experience from the graduates’ perspective.

## Supplementary Information



## Figures and Tables

**Table 1:** Demographics

Gender (N=34)	N	Percent (%)
Female	16	47
Male	18	53

**Age (N=35)**		

25–34 years old	16	46
35–44 years old	15	43
45–54 years old	1	3
55–64 years old	3	9

**Specialty (N=35)**		

Emergency Medicine	25	71
Anesthesiology	5	14
Surgery	3	9
Nursing	2	6

**Length of simulation fellowship (N=35)**		

< 1 year	2	6
1 year	27	77
2 years	6	17

**Number of years since completion of simulation fellowship (N=35)**		

< 1 year	13	37
1 year	0	0
2 years	10	29
3–5 years	9	26
> 5 years	3	9

**Table 2:** Simulation fellowship experience

Fellowship time distribution (average time per week)	N	Percent (%)
**Simulation fellowship responsibilities (N = 33)**		

1–10 hours/week	2	6
11–20 hours/week	12	36
21–30 hours/week	9	27
31–40 hours/week	7	21
>40 hours/week	3	9

**Clinical fellowship responsibilities (N=34)**		

1–10 hours/week	7	21
11–20 hours/week	21	62
21–30 hours/week	6	18
31–40 hours/week	0	0
>40 hours/week	0	0

**Number of months into fellowship you felt you had the ability to effectively debrief with minimal guidance (N=34)**		

1–3 months	9	26
4–6 months	15	44
7–10 months	7	21
10–12 months	2	6
More than 12 months	1	3
I could not by the end of fellowship	0	0

**Number of months into fellowship you felt you had the ability to run a high-fidelity full body simulator with minimal guidance (N=34)**		

1–3 months	20	59
4–6 months	6	18
7–10 months	3	9
10–12 months	4	12
More than 12 months	0	0
I could not by the end of fellowship	1	3
Fellows did not run simulators	0	0

**Table 3:** Preparation for post-fellowship stimulation career

	N	Percent (%)
**Fellowship adequately prepared me for post-fellowship career**		

Strongly Agree	20	59
Agree	10	29
Neither Agree Nor Disagree	4	12
Disagree	0	0
Strongly Disagree	0	0
Minimal Involvement post-fellowship	0	0

**Research experience in fellowship adequately prepared my for post-fellowship career**		

Strongly Agree	8	24
Agree	15	44
Neither Agree Nor Disagree	7	21
Disagree	3	9
Strongly Disagree	0	0
Minimal Involvement post-fellowship	1	3

**Simulation technology training adequately prepared my for post-fellowship career**		

Strongly Agree	9	26
Agree	20	59
Neither Agree Nor Disagree	3	9
Disagree	1	3
Strongly Disagree	0	0
Minimal Involvement post-fellowship	1	3

**Debriefing training adequately prepared my for post-fellowship career**		

Strongly Agree	26	76
Agree	8	24
Neither Agree Nor Disagree	0	0
Disagree	0	0
Strongly Disagree	0	0
Minimal Involvement post-fellowship	0	0

**Administrative training adequately prepared my for post-fellowship career**		

Strongly Agree	7	21
Agree	14	42
Neither Agree Nor Disagree	6	18
Disagree	2	6
Strongly Disagree	1	3
Minimal Involvement post-fellowship	3	9

**Faculty feedback/mentorship was adequate**		

Strongly Agree	18	53
Agree	12	35
Neither Agree Nor Disagree	3	9
Disagree	1	3
Strongly Disagree	0	0
Minimal Involvement post-fellowship	n/a	n/a

## Appendix A Simulation fellowship programs surveyed

**Table:**

Canadian Simulation Fellowships	Location
McGill University	Montreal, QC
Mount Sinai Hospital (Toronto)	Toronto, ON
St. Michaels Hospital (Toronto)	Toronto, ON
Sunnybrook Health Sciences Centre (Toronto)	Toronto, ON
University of Ottawa	Ottawa, ON
University of Western Ontario	London, ON

**United States Simulation Fellowships**	**Location**

Akron City Hospital	Akron, OH
Alpert Medical School of Brown University	Providence, RI
Drexel University College of Medicine	Philadelphia, PA
John H. Stroger of Cook County Hospital	Chicago, IL
Johns Hopkins	Baltimore, MD
Maimonides Medical Center	New York, NY
Massachusetts General Hospital	Boston, MA
STRATUS Center for Medical Simulation at Brigham and Women’s Hospital	Boston, MA
New York University Langone Medical Center	New York, NY
North Shore University Health System	Evanston, IL
St. Luke’s-Roosevelt Hospital Center New York	New York, NY
Stanford University School of Medicine	Stanford, CA
St. Louis University	St. Louis, MO
SUNY Downstate/Kings County Hospital	New York, NY
University of California Davis	Davis, CA
University of California Irvine	Irvine, CA
University of California-Irvine Anesthesia	Irvine, CA
University of Illinois at Chicago	Chicago, IL
University of Minnesota	Minneapolis, MN
University of Virginia	Charlottesville, VA
Veterans Affairs Hospitals	Orlando, FL
Yale University/Yale New Haven Hospital	New Haven, CT

## Appendix B Simulation fellowship graduate survey

See eSupplement 1

## References

[1] Passiment M, Sacks H, Huang G (2011). Medical Simulation in Medical Education: Results of an AAMC survey.

[2] Barsuk JH, Cohen ER, McGaghie WC, Wayne DB (2010). Long-term retention of central venous catheter insertion skills after simulation-based mastery learning. Acad Med.

[3] Wayne DB, Didwania A, Feinglass J, Fudala MJ, Barsuk JH, McGaghie WC (2008). Simulation based education improves quality of care during cardiac arrest team responses at an academic teaching hospital: a case– control study. Chest.

[4] Wayne DB, Butter J, Siddall VJ (2006). Mastery learning of advanced cardiac life support skills by internal medicine residents using simulation technology and deliberate practice. J Gen Intern Med.

[5] Kessler DO, Auerbach M, Pusic M, Tunik MG, Foltin JC (2011). A randomized trial of simulation-based deliberate practice for infant lumbar puncture skills. Simul Healthc.

[6] Zendejas B, Cook DA, Hernandez-Inizarry R, Huebner M, Farley DR (2012). Mastery learning simulation-based curriculum for laparoscopic TEP inguinal hernia repair. J Surg Educ.

[7] Levine AI, DeMaria S, Schwartz AD, Sim AJ (2013). The comprehensive textbook of healthcare simulation.

[8] Okuda Y, Bryson EO, DeMaria S (2009). The Utility of Simulation in Medical Education: What is the evidence?. Mt Sinai J Med.

[9] Okuda Y, Bond W, Bonfante G (2008). National growth in simulation training within emergency medicine residency programs, 2003–2008. Acad Emerg Med.

[10] Ahmed RA, Frey J, Gardner AK, Gordon JA, Yudkowsky R, Tekian A (2016). Characteristics and core curricular elements of medical simulation fellowships in North America. J Grad Med Educ.

[11] Stern S (2002). Fellowship training: a necessity in today’s academic world. Acad Emerg Med.

[12] Taylor JS, Friedman RH, Speckman JL, Ash AS, Moskowitz MA, Carr PL (2001). Fellowship training and career outcomes for primary care physician-faculty. Acad Med.

[13] Broaddus VC, Feigal DW (1994). Starting an academic career. A survey of junior academic pulmonary physicians. Chest.

[14] Santen SA, Deiorio NM, Gruppen LD (2012). Medical education research in the context of translational science. Acad Emerg Med.

[15] Cohen ER, Feinglass J, Barsuk JH (2010). Cost savings from reduced catheter-related bloodstream infection after simulation-based education for residents in a medical intensive care unit. Simul Healthc.

[16] Barsuk JH, Cohen ER, Feinglass J, McGaghie WC, Wayne DB (2011). Unexpected collateral effects of simulation-based medical education. Acad Med.

[17] Ahmed RA (2015). Characteristics and Core Curricular Elements of Medical Simulation Fellowships in North America Master of Health Professions Education [thesis].

[18] Kern D, Thomas P, Hughes M (2009). Curriculum Development for Medical Education: A Six-Step Approach.

[19] McGaghie W, Issenberg S, Petrusa E, Scalese R (2010). A critical review of simulation-based medical education research: 2003–2009. Med Educ.

[20] National Resident Matching Program (2015). Results and Data 2015 Main Residency Match.

