# Supplementary material for: Training the trainers: a survey of simulation fellowship graduates
Source: Can Med Educ J. 2017 Jun 30;8(3):e81–9. (PMC5661740)
Supplement: Supplementary file 1 [file CMEJ-08-81-s001.pdf]

Simulation Fellow Survey

Survey Introduction

Dear Simulation Fellowship Graduate,

You are invited to participate in a research study entitled, “Training the trainers: A survey of simulation fellowship graduates.” The overarching objective of this study is to evaluate your simulation fellowship experience and how the fellowship prepared you for your post-fellowship career.

I am an emergency medicine attending physician and simulation fellow. This study will serve as my fellowship research project. Given the small number of graduates, your participation is very important for the accurate reflection of the simulation fellowship experience.

This survey is voluntary and anonymous. It should take approximately 5 minutes to complete. All questions are optional to answer. If you have previously participated in this survey, please do not take the survey again.

While you will not experience any direct benefits from participation, the information collected will benefit current and future simulation fellows by providing an improved understanding of the fellow experience.

The Institutional Review Board of Summa Akron City Hospital has approved this survey. There are no known risks associated with participation in this study. All information will be collected and reported anonymously.

This study is being conducted by the Department of Emergency Medicine at Summa Akron City Hospital.

If you have any questions about your rights as a research subject you may call the Summa Health System Institutional Review Board (IRB) at 330-375-4045. The IRB is a group of people who assure that appropriate steps are taken to protect the rights and welfare of humans participating as subjects in a research study.

If you have any questions about this study or would like to send me any documents, please contact me at hughesp@summahealth.org

Clicking on next below implies consent for participation in this study.

Thank you for your participation.

Patrick G. Hughes, D.O.
Medical Simulation Fellow
Summa Health System
525 E. Market Street
Akron, OH 44304
Phone: (330) 375-3604

Simulation Fellow Survey

General Information

How many years since you completed your simulation fellowship?

- ◯ Less than one year ago
- ◯ One year ago
- ◯ Two years ago
- ◯ Three to five years ago
- ◯ More than five years ago

How long was your simulation fellowship?

- ◯ Less than one year
- ◯ One year
- ◯ Two years
- ◯ Other (please specify)

What is your specialty? (Check all that apply)

- ☐ Emergency Medicine
- ☐ Surgery
- ☐ Anesthesiology
- ☐ Internal Medicine
- ☐ Pediatrics
- ☐ Obstetrics/Gynecology
- ☐ Family Medicine
- ☐ Nursing
- ☐ Other (please specify)

Simulation Fellow Survey

Demographic Information

What is your age?

- ◯ 18 to 24 years
- ◯ 25 to 34 years
- ◯ 35 to 44 years
- ◯ 45 to 54 years
- ◯ 55 to 64 years
- ◯ 65 to 74 years
- ◯ 75 years or older

What is your gender?

- ◯ Female
- ◯ Male

Simulation Fellowship Experience - Work Time Distribution

As a fellow, what was the average time per week spent on simulation fellowship responsibilities?

- ◯ 1 - 10 hours per week
- ◯ 11 - 20 hours per week
- ◯ 21 - 30 hours per week
- ◯ 31 - 40 hours per week
- ◯ More than 40 hours per week

As a fellow, what was the average time per week spent on clinical responsibilities (ie. shifts, clinic hours)?

- ◯ 1 - 10 hours per week
- ◯ 11 - 20 hours per week
- ◯ 21 - 30 hours per week
- ◯ 31 - 40 hours per week
- ◯ More than 40 hours per week

As a fellow, what percent of your time was spent on the following fellowship activities (responses should total 100%):

Teaching/debriefing learners	
Simulation technical/technology training (includes learning to use task trainers, operating high-fidelity simulators, etc)	
Simulation curriculum development (e.g. scenario design, etc.)	
Research design & reporting	
Administration	
Other	

Please describe other fellowship activities.

Simulation Fellow Survey

Fellowship Experience - Achievements and Professional Development

How many scholarly projects did you complete during your fellowship? (Choose all that apply)

	0	1	2	3	4	≥5
Local/Regional Presentation/Abstract	○	○	○	○	○	○
National Presentation/Abstract	○	○	○	○	○	○
International Presentation/Abstract	○	○	○	○	○	○
Full Manuscript Submissions (does not have to be accepted for publication during fellowship)	○	○	○	○	○	○

Please describe other scholarly projects and number completed (e.g. patents, published simulation cases):

How many months into the fellowship did you feel that you had the ability to conduct effective debriefing with minimal guidance?

- ○ 1 - 3 months
- ○ 4 - 6 months
- ○ 7 - 9 months
- ○ 10 - 12 months
- ○ More than 12 months
- ○ I could not conduct an effective debriefing with minimal guidance by the end of fellowship

How many months into the fellowship did you feel that you had the ability to utilize/run a high-fidelity full body simulator effectively with minimal guidance?

- [ ] 1 - 3 months
- [ ] 4 - 6 months
- [ ] 7 - 9 months
- [ ] 10 - 12 months
- [ ] More than 12 months
- [ ] I could not run a high-fidelity simulator effectively with minimal guidance by the end of fellowship
- [ ] Fellows did not run the simulators

Did you pursue a masters or advanced degree during your fellowship?

- ○ Yes
- ○ No

Simulation Fellow Survey

Degree Obtained During Fellowship

What degree did your pursue/earn during your simulation fellowship? (Choose all that apply)

- [ ] Masters in Education/Medical Education (MEd)
- [ ] Masters in Health Administration (MHA)
- [ ] Masters in Public Health (MPA)
- [ ] Masters in Business Administration (MBA)
- [ ] Doctorate of Philosphy (Ph.D.)
- [ ] Other (please specify)

Simulation Fellow Survey

Simulation Fellowship Experience - Quality of the Training Received

My simulation fellowship adequately prepared me for my post-fellowship simulation career.

Strongly Agree	Agree	Neither Agree Nor Disagree	Disagree	Strongly Disagree	Minimal Involvement in Simulation Education Post Fellowship
○	○	○	○	○	○

The research experience in my fellowship adequately prepared me to conduct research in my post-fellowship simulation career.

Strongly Agree	Agree	Neither Agree Nor Disagree	Disagree	Strongly Disagree	Minimal Involvement in Research Post Fellowship
○	○	○	○	○	○

The simulation technology training (running / troubleshooting simulator, simulation environment set up) in my fellowship adequately prepared me for my post-fellowship simulation career?

Strongly Agree	Agree	Neither Agree Nor Disagree	Disagree	Strongly Disagree	Minimal Involvement in Simulation Technology Post Fellowship
○	○	○	○	○	○

The debriefing training in my fellowship adequately prepared me for my post-fellowship simulation career.

Strongly Agree	Agree	Neither Agree Nor Disagree	Disagree	Strongly Disagree	Minimal Involvement in Debriefing Post Fellowship
○	○	○	○	○	○

The administrative training in my fellowship adequately prepared me for my post fellowship simulation career.

Strongly Agree	Agree	Neither Agree Nor Disagree	Disagree	Strongly Disagree	Minimal Involvement in Administration Post Fellowship
○	○	○	○	○	○

Faculty feedback/ mentorship during my fellowship was adequate.

Strongly Agree	Agree	Neither Agree Nor Disagree	Disagree	Strongly Disagree
○	○	○	○	○

Simulation Fellow Survey

Post - Simulation Fellowship Experience

What was the most challenging part of YOUR fellowship curriculum to master? (Choose one)

- ○ Teaching/debriefing learners
- ○ Simulation technical/technology training (includes learning to use task trainers, operating high-fidelity simulators, etc)
- ○ Simulation curriculum development (e.g. scenario design, etc.)
- ○ Research design & reporting
- ○ Administration

Other or Explanation for above answer

What area in your fellowship's curriculum needed the most improvement? (Choose one)

- ○ Teaching/debriefing learners
- ○ Simulation technical/technology training (includes learning to use task trainers, operating high-fidelity simulators, etc)
- ○ Simulation curriculum development (e.g. scenario design, etc.)
- ○ Research design & reporting
- ○ Administration
- ○ Other (please specify)

IMMEDIATELY after completion of your simulation fellowship, what was your next professional appointment?

- Simulation Director
- Simulation Assistant Director
- Simulation Faculty Appointment (Non–director)
- Faculty Appointment/Clinical Faculty (No Stimulation)
- Independent Clinical Work (No Simulation)
- Other (please specify)

Simulation Fellow Survey

Post - Simulation Fellowship Experience - Current Role

Are you ***CURRENTLY*** a Simulation Director, Assistant Simulation Director or actively involved in simulation education?

- ○ Yes
- ○ No

Simulation Fellow Survey

Post - Simulation Fellowship Experience - Current Role

What is your ***CURRENT*** professional appointment?

- Simulation Director
- Simulation Assistant Director
- Simulation Faculty (Non–Director)
- Other (please specify)

What is your ***CURRENT*** simulation center's affiliation?

- University/Academic Center affiliated
- Free Standing (no affiliation)
- Community Hospital affiliated
- Government affiliated
- Other (please specify)

What is the number of visits / learner encounters at your ***CURRENT*** simulation center annually?

- Less than 1,000 visits per year
- 1,000 - 5,000 visits per year
- 5,000 - 10,000 visits per year
- More than 10,000 visits per year

What is ***<u>your full time equivalents (FTE's)</u>*** protected time for simulation education in your***<u>CURRENT</u>*** job?

- ◯ 0.00 (volunteer)
- ◯ 0.25
- ◯ 0.5
- ◯ 0.75
- ◯ 1.0
- ◯ Other (please specify)

Simulation Fellow Survey

Additional Comments

What was the most difficult aspect of your transition from a medical simulation fellow into your ***FIRST*** simulation based role?

Is there any other information you would like to share in regards to your Simulation Fellowship Experience?
